# Development of a Double-Antibody Sandwich ELISA Based on a Monoclonal Antibody against the Viral NS1 Protein for the Detection of Chicken Parvovirus

**DOI:** 10.3390/pathogens13030221

**Published:** 2024-03-01

**Authors:** Minxiu Zhang, Jianqi Liao, Zhixun Xie, Yanfang Zhang, Sisi Luo, Meng Li, Liji Xie, Qing Fan, Tingting Zeng, Jiaoling Huang, Sheng Wang

**Affiliations:** Key Laboratory of China (Guangxi)-ASEAN Cross-Border Animal Disease Prevention and Control, Ministry of Agriculture and Rural Affairs, Guangxi Key Laboratory of Veterinary Biotechnology, Guangxi Veterinary Research Institute, Nanning 530001, China; zhminxiu2010@163.com (M.Z.); li_aojianqi@126.com (J.L.); zhangyanfang409@126.com (Y.Z.); 2004-luosisi@163.com (S.L.); mengli4836@163.com (M.L.); xie3120371@163.com (L.X.); fanqing1224@126.com (Q.F.); tingtingzeng1986@163.com (T.Z.); huangjiaoling728@126.com (J.H.); wangsheng1021@126.com (S.W.)

**Keywords:** chicken parvovirus, NS1 protein, monoclonal antibodies, DAS-ELISA

## Abstract

Chicken parvovirus (ChPV) infection can cause runting-stunting syndrome (RSS) in chickens. There is currently no commercially available vaccine for controlling ChPV, and ChPV infection in chickens is widespread globally. The rapid detection of ChPV is crucial for promptly capturing epidemiological data on ChPV. Two monoclonal antibodies (mAbs), 1B12 and 2B2, against the ChPV NS1 protein were generated. A double-antibody sandwich enzyme-linked immunosorbent assay (DAS-ELISA) was developed for detecting ChPV based on the mAb 1B12 and an anti-chicken polyclonal antibody against the ChPV NS1 protein. The detection limit for the ChPV recombinant pET32a-NS1 protein was approximately 31.2 ng/mL. A total of 192 throat and cloaca swab samples were analyzed for ChPV by the established DAS-ELISA and nested PCR methods. The concordance rate between the DAS-ELISA and the nested PCR method was 89.1%. The DAS-ELISA can detect the ChPV antigen without any cross-reaction with FAdV-4, FAdV-1, NDV, AIV, MS, CIAV, aMPV, EDSV, IBV, or AGV2. The method also has high repeatability, with a coefficient of variation (CV) of less than 5%. These findings indicate that the DAS-ELISA exhibits high accuracy, good sensitivity, and specificity, making it suitable for viral detection, field surveillance, and epidemiological studies.

## 1. Introduction

Chicken parvovirus (ChPV) is a nonenveloped, single-stranded DNA virus that belongs to the genus *Aveparvovirus* within the subfamily *Parvovirinae* of the family *Parvoviridae* [1]. ChPV was first discovered in the feces of young chickens with runting-stunting syndrome (RSS) in Hungary in 1984 [2]. ChPV can cause watery diarrhea and growth retardation in broiler chicks [3,4]. The size of the ChPV genome is approximately 5 kb, and it contains three open reading frames (ORFs) that encode four proteins: two structural proteins (VP1 and VP2) and two nonstructural proteins (NS1 and NP1) [5]. The VP1 protein is composed of 671 amino acids and may play an essential role in the process of ChPV entering cells and eventually releasing viruses. The VP2 protein is a capsid protein composed of 537 amino acids and is associated with functions such as DNA replication and virus packaging [6]. The NP1 protein contains approximately 101 amino acids. However, the function of the NP1 protein in ChPV is unclear. Previous studies have shown that the NP1 protein is a nonstructural protein necessary for the efficient replication of viral DNA and control of capsid protein expression in human Boca virus, which also belongs to the same family, *Parvoviridae* [7]. NS1 is the most important nonstructural protein and consists of 694 amino acids. This protein is a nuclear phosphoprotein that is primarily located in the cell nucleus and is involved in viral replication and assembly [8].

The pathological characteristics and clinical symptoms of ChPV-infected chickens are similar to those of chickens infected by chicken astrovirus (CAstV), avian rotavirus (AvRV), and picornavirus [9,10,11], leading to difficulties in differential diagnosis based on clinical features. Therefore, it is necessary to develop a detection method for identifying ChPV. The *NS1* gene is highly conserved among chicken parvoviruses and is often used as a target gene for the detection of ChPV nucleic acids [12,13]. Currently, polymerase chain reaction (PCR) and real-time PCR (RT-qPCR) are two commonly used methods for determining the presence of ChPV [12,13]. However, serological assays for detecting ChPV are rare. Enzyme-linked immunosorbent assays (ELISAs) are simple and cost-efficient serological assays that do not require viral DNA or RNA extraction. ELISA-based methods have been developed for pathogen detection [14,15,16].

RSS is an enteric disease in young poultry characterized by clinical symptoms such as diarrhea, depression, decreased weight gain, and growth delay, causing significant economic losses in the poultry industry [17]. The etiological agents that cause RSS are complex. The occurrence of RSS in poultry has been described as possibly being related to infection by one or more poultry enteroviruses, including poultry parvovirus, CAstV, AvRV, picornavirus, avian reovirus (ARV), and infectious bronchitis virus (IBV) [9,10,11,18,19,20]. ChPV has been detected in chickens with RSS in a few countries, such as India, Brazil, Korea, Poland, and China [21,22,23,24,25]. Zsak et al. [4] and Nuñez et al. [26] showed that SPF chicks infected with ChPV exhibit obvious clinical symptoms of RSS. In addition, ChPV infections are prevalent in healthy chickens [27]. Currently, there is no vaccine available to prevent or control ChPV infections, so it is essential to detect the virus to evaluate the impact of ChPV infections.

In this study, two monoclonal antibodies (mAbs) targeting the NS1 protein of ChPV were generated, and a double-antibody sandwich ELISA (DAS-ELISA) was used to detect ChPV based on a mAb and polyclonal antibody. The established DAS-ELISA was sensitive and specific for detecting ChPV infection, providing a new tool for ChPV surveillance.

## 2. Materials and Methods

### 2.1. Cells, Clinical Samples and Viruses

SP2/0 myeloma cells and chicken liver cancer cells (LMHs) were preserved by the Guangxi Veterinary Research Institute (Gaungxi, China); 50 ChPV-negative throat and cloacal swab samples were collected from specific pathogen-free (SPF) chickens and used to determine the cut-off value; and 192 throat and cloacal swab samples were collected from chickens in live poultry markets in Guangxi, China.

Newcastle disease virus (NDV), *Mycoplasma gallisepticum* (MS), fowl adenovirus serotype 4 (FAdV-4), fowl adenovirus serotype 1 (FAdV-1), chicken infectious anemia virus (CIAV), chicken infectious bronchitis virus (IBV), avian metapneumonia virus (aMPV), H9N2 subtype avian influenza virus (AIV), avian egg drop syndrome virus (EDSV), and an avian circovirus 2 (AGV2)-positive throat and cloacal swab sample were preserved in the laboratory and used for testing the specificity of the DAS-ELISA. The details of these pathogens are shown in Table 1.

### 2.2. Full-Length Infectious Plasmid of ChPV, Recombinant pET32a-NS1 Protein, and Polyclonal Antibodies

The full-length infectious plasmid of ChPV (pBluescript II SK (+)-ChPV), which contains the whole ORF of the ChPV strain GX-CH-PV-21 (GenBank: MG602511), was transfected into LMH cells (to obtain the ChPV infectious clone) for use in the specific DAS-ELISA experiment. LMH cells infected with the pBluescript II SK (+)-ChPV plasmid were also subjected to immunofluorescence analysis (IFA) and Western blotting (WB). The purified recombinant pET32a-NS1 protein (104 kDa) containing the whole NS1 protein from strain GX-CH-PV-21 was preserved at −80 °C before use for animal immunity and WB analysis with mAbs. The purified polyclonal antibodies (1.5 mg/mL) were preserved at −80 °C and produced by immunizing SPF chickens with recombinant pET32a-NS1 protein [28].

### 2.3. Production and Identification of mAbs against the ChPV-NS1 Protein

Eight-week-old BALB/c mice were immunized four times with purified recombinant pET32a-NS1 protein. The mice were subcutaneously immunized with 100 µg/mouse recombinant pET32a-NS1 protein emulsified with complete Freund’s adjuvant (Sigma-Aldrich, St. Louis, MO, USA) at the first immunization. The second and third immunizations were performed with the same dose of recombinant pET32a-NS1 protein emulsified with incomplete Freund’s adjuvant at 21 and 35 days after the first immunization. After 49 days, the antibody titers against the recombinant pET32a-NS1 protein in the immunized mice were determined via indirect ELISA. Mice with high antibody titers were immunized with 1.0 mL of recombinant pET32a-NS1 protein solution (400 µg/mL) without adjuvant via intraperitoneal injection at 56 days (the fourth immunization). On the third day after the fourth immunization, the splenocytes of the mice were harvested. The fusion of splenocytes with SP2/0 myeloma cells was performed according to methods previously described by Luo et al. [29]. Positive hybridoma cells with high antibody titers against the recombinant pET32a-NS1 protein were screened by indirect ELISA. The positive hybridoma cells were subcloned three times, and the antibody titers of the hybridoma supernatants of each subclone were determined using an indirect ELISA. Finally, positive hybridoma cells with high antibody titers were selected and injected into the abdominal cavities of the mice. The ascitic fluid secreted after the injection of positive hybridoma cells was harvested and purified according to methods previously described by Wang et al. [14]. The mAb isotypes were determined using a commercial mouse mAb isotyping kit (Sigma-Aldrich, St. Louis, MO, USA).

The steps of the indirect ELISA mentioned above were as follows: (1) 96-well microtiter plates were coated with 100 µL/well recombinant pET32a-NS1 protein at a concentration of 5 μg/mL in phosphate-buffered saline (PBS) and then incubated at 37 °C for 1 h. (2) The plates were subsequently blocked with blocking buffer (5% skim milk powder in PBS) at 37 °C for 1 h. (3) The plates were washed three times with PBS containing 0.1% Tween-20 (PBST). (4) Serum samples from immunized mice or the supernatant of hybridoma cells were added to the wells of the plates. Serum samples from nonimmunized mice were used as negative controls. The plates were incubated at 37 °C for 1 h and then washed again. (5) HRP-labelled goat anti-mouse IgG (Beyotime Biotechnology Co., Ltd., Shanghai, China) (100 µL/well) at a dilution of 1:2000 was added to each well, and the plates were incubated at 37 °C for 45 min. (6) After a washing step, 3,3′,5,5′-tetramethylbenzidine (TMB) solution (100 µL/well) was added, and the plates were incubated in the dark at room temperature for 10 min. (7) Then, 100 µL/well sulfuric acid (2 M) was added, and the absorbance at 450 nm was measured immediately using an ELISA plate reader (Shanghai Kehua Bio-Engineering Co., Ltd., Shanghai, China). When the OD_450nm_ of the sample/OD_450nm_ value of the negative control was greater than 2.1, the sample was considered positive.

### 2.4. WB Analysis and IFA

WB analysis and IFA were performed to determine the reactivity and specificity of the mAbs against the recombinant pET32a-NS1 protein and the NS1 protein expressed in the LMH cells. (1) LMH cells were transfected with the plasmid pBluescript II SK (+)-ChPV. After 3 days, the LMH cells were collected and lysed for SDS–PAGE. The NS1 protein expressed in LMH cells was approximately 79 kDa in length. (2) In addition, the purified recombinant pET32a-NS1 protein (104 kDa) was subjected to SDS–PAGE. (3) Then, the proteins in the gel were transferred onto a polyvinylidene fluoride (PVDF) membrane. The membrane was blocked with 5% skim milk overnight at 4 °C. The membrane was subsequently washed three times with PBST. (4) Primary antibodies (mAbs) (1:1000 dilution) against the NS1 protein harvested as described in Section 2.3 were added to the membrane and incubated at 37 °C for 1 h. (5) The membrane was washed three times. Then, alkaline phosphatase (AP)-labelled goat anti-mouse IgG (1:2000 dilution) was used as a secondary antibody and was added, and the membrane was incubated at 37 °C for 1 h. (6) The membrane was washed and then stained with a commercial BCIP/NBT alkaline phosphatase color development kit (Beyotime Biotechnology Co., Ltd., Shanghai, China).

LMH cells cultured in 6-well plates were transfected with the plasmid pBluescript II SK (+)-ChPV. LMH cells were fixed with 4% paraformaldehyde three days after transfection. The mAbs (1:500 dilution) were incubated with LMH cells at 37 °C for 1 h. A FITC-labelled goat anti-mouse IgG antibody (1:500) was added after the cells were washed three times with PBST, and the plate was incubated at 37 °C for 1 h. The LMH cells were subsequently washed again and observed via fluorescence microscopy.

### 2.5. Selection of the Capture Antibody

The mAbs were used as the capture antibodies and were coated on a 96-well microtiter plate to determine the optimal capture antibody by the DAS-ELISA. For this purpose, 1:1000 dilutions of 2.0 mg/mL stocks of purified mAbs were coated (100 μL/well) on a 96-well microtiter plate. The recombinant pET32a-NS1 protein in PBS (5 μg/mL) and the cell suspension of the ChPV infectious clone were used as the sandwich antigen, and the polyclonal antibody against the NS1 protein from the SPF chickens was used as the detection antibody. PBS and a cell suspension of negative LMH cells were used as negative controls. Each sample was tested in triplicate. The absorbance at 450 nm of the mixture in the wells of the plate was measured. If the OD_450nm_ value of the sample/OD_450nm_ value of the negative control was greater than 2.1, the sample was considered positive.

### 2.6. Development of an NS1-DAS-ELISA for ChPV Detection

The purified mAb and polyclonal antibody were used as the capture and detection antibodies, respectively. The optimal concentrations of the mAbs and polyclonal antibodies were determined via checkerboard titration. The steps of NS1-DAS-ELISA were as follows: (1) 1:1000, 1:2000, 1:4000, 1:8000, 1:16,000, and 1:32,000 dilutions of 2.2 mg/mL stocks of mAbs were coated (100 μL/well) on the 96-well microtiter plate at 4 °C for 18 h. The plate was subsequently blocked with blocking buffer (5% skim milk powder in PBS) at 37 °C for 45 min. (2) Then, the plate was washed three times with PBST, after which, 100 µL/well recombinant pET32a-NS1 protein in PBS (5 μg/mL) was added. (3) According to the checkerboard titration, 1:1000, 1:2000, 1:4000, 1:8000, 1:16,000, 1:32,000, 1:64,000, and 1:128,000 dilutions of 1.5 mg/mL stocks of polyclonal antibodies were added to each well after washing with PBST, and the plate was then incubated at 37 °C for 45 min. (4) The plate was washed again. HRP-labelled goat anti-chicken IgG (Beyotime Biotechnology Co., Ltd., Shanghai, China) (100 µL/well) at a dilution of 1:2000 was added, and the mixture was incubated at 37 °C for 45 min. (5) Then, the wells were washed with PBST, and TMB solution (100 µL/well) was added. The mixture was incubated in the dark for 11 min at 25 °C. The color reaction was stopped with 100 µL of sulfuric acid (2 M, 100 µL/well), after which the absorbance at 450 nm was measured. (6) The ideal concentrations of the capture antibody and detection antibody were determined according to the above 5 steps. Then, the concentration of HRP-labelled goat anti-chicken IgG (diluted 1:500, 1:1000, 1:2000, 1:4000 and 1:8000) was further optimized.

### 2.7. NS1-DAS-ELISA Positive and Negative Cut-Off Values

A total of 50 ChPV-negative throat and cloacal swab samples were collected from SPF chickens. These samples were analyzed by NS1-DAS-ELISA, and the OD_450nm_ values of 50 samples were obtained. The critical value was $\bar{x}$ + 3SD (where “$\bar{x}$” represents the mean OD_450nm_ value of 50 samples, and “SD” represents the standard deviation).

### 2.8. Specificity and Sensitivity of the NS1-DAS-ELISA

The specificity of the NS1-DAS-ELISA was tested using suspensions of LMH cells transfected with pBluescript II SK (+)-ChPV, NDV, MS, FAdV-1, FAdV-4, CIAV, IBV, aMPV, H9N2 subtype AIV, EDSV, and AGV2. Each pathogen was tested in triplicate.

Due to the lack of available ChPV isolates and the low viral titer of the ChPV infectious clones in the LMH cells, the recombinant pET32a-NS1 protein was used for sensitivity analysis. The recombinant pET32a-NS1 protein was diluted to concentrations of 1000, 500, 250, 125, 62.5, 31.2, 15.6, 7.8, and 0 ng/mL with PBS. The sensitivity of the NS1-DAS-ELISA was evaluated with different recombinant pET32a-NS1 protein concentrations (100 µL/well), and each concentration of the recombinant pET32a-NS1 protein was tested in triplicate.

### 2.9. Repeatability Analysis of the NS1-DAS-ELISA

The repeatability of the NS1-DAS-ELISA was tested using different concentrations of the recombinant pET32a-NS1 protein (at dilutions of 250 ng/mL, 500 ng/mL, 750 ng/mL, and 1000 ng/mL), and the ChPV-negative sample from SPF chickens was used as a negative control. The intra- and interassay repeatability tests were performed using the same batch of the ELISA plates or three different batches of the ELISA plates, respectively. The same concentration of the recombinant pET32a-NS1 protein and the negative control samples were tested in triplicate. The means of the OD_450nm_ values, standard deviations, and percent coefficients of variation (% CVs) were calculated with SPSS software version 22.0 (IBM SPSS Inc., Chicago, IL, USA).

### 2.10. Comparison of the NS1-DAS-ELISA and Nested PCR Methods

The established NS1-DAS-ELISA and a nested PCR method [30] were used to analyze 192 throat and cloacal swab samples. The primers used for nested PCR were designed based on the *NS1* gene. For nested PCR, the primer pair used in the first round of amplification were 661F (5′-GGTACAAGATATGCTAGATTT-3′) and 1073R (5′-CGGATGGCTAAATTATCATCT-3′). In the second round, the primer pair used was 718F (5′-CCATCGCAGGAATTAACTCCAG-3′) and 1043R (5′-GTGTCAACATCTCCATGTATTG-3′). The first and second rounds of the nested PCR amplification procedure were as follows: 95 °C for 3 min; 30 cycles of 95 °C for 1 min, 53 °C for 30 s, and 72 °C for 1 min; and 72 °C for 5 min. The detection results of the two methods were comparatively analyzed.

## 3. Results

### 3.1. Production and Identification of mAbs against the ChPV-NS1 Protein

Two positive hybridoma cell lines capable of secreting mAbs against the NS1 protein, namely, 1B12 and 2B2, were obtained. The titers of the mAbs (1B12 and 2B2) in the mouse ascites fluid were 1:1.6 × 10^7^ and 1:4.1 × 10^6^, respectively. The isotyping assay showed that 1B12 and 2B2 are IgG1 with κ light chains (Table 2).

### 3.2. WB Analysis and IFA of the mAbs

**Figure 1:**
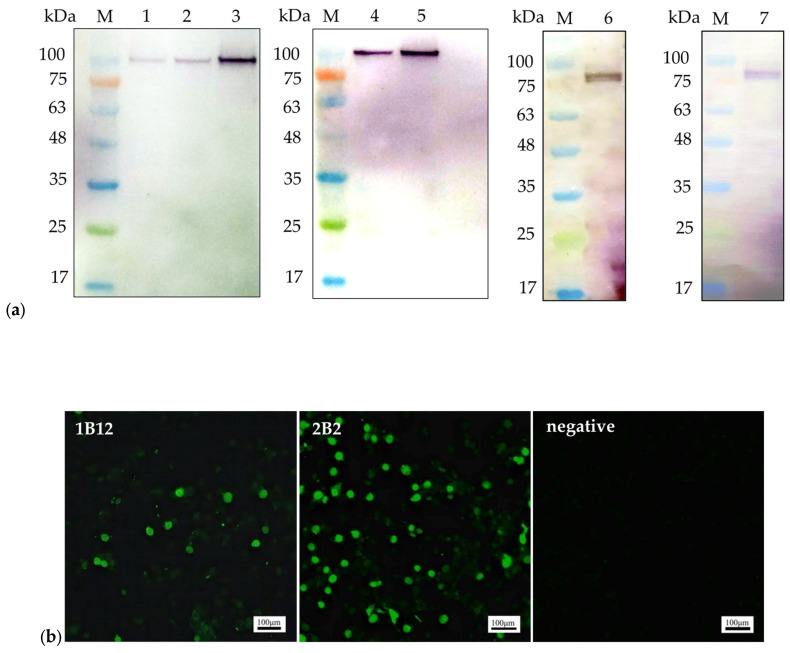
The reactivity of the monoclonal antibodies: (**a**). Western blot analysis of the mAbs. mAb 1B12 (lane 1, lane 2, and lane 3) reacted with the 104 kDa recombinant pET32a-NS1 protein; mAb 2B2 (lane 4 and lane 5) reacted with the 104 kDa recombinant pET32a-NS1 protein. The mAbs 1B12 and 2B2 (lane 6 and lane 7) reacted with the 79 kDa NS1 protein expressed in LMH cells; (**b**). IFA was performed on LMH cells transfected with the ChPV infectious plasmid pBluescript II SK(+)-ChPV. Scale bars, 100 µm.

The binding abilities of mAbs 1B12 and 2B2 were verified using WB and IFA. The NS1 proteins used in the WB analysis were the recombinant pET32a-NS1 protein (104 kDa) expressed in *Escherichia coli* and NS1 protein (79 kDa) expressed in LMH cells (Figure 1a). IFA was performed to detect NS1 protein expression in LMH cells transfected with the ChPV infectious plasmid pBluescript II SK(+)-ChPV (Figure 1b).

### 3.3. Selection of the Capture Antibody

The mAbs 1B12 and 2B2 were used as the capture antibodies in the DAS-ELISA. The DAS-ELISA was performed to determine the optimal capture antibody. The results are shown in Table 3. The mAb 1B12 had a higher OD_450nm_ value than the mAb 2B2 (Table 3), and the titer of the mAb 1B12 was also greater than that of the mAb 2B2 (see Section 3.1). Therefore, the mAb 1B12 was chosen as the capture antibody for the development of the NS1-DAS-ELISA.

### 3.4. Development of the NS1-DAS-ELISA

The optimal concentrations of the capture antibody, detection antibody, and HRP-labelled goat anti-chicken IgG were as follows: a 1:2000 dilution of a 2.2 mg/mL stock of mAbs was applied to the ELISA plate, and the mixture was incubated at 4 °C for 18 h. Polyclonal antibodies (1:4000 dilutions of 1.5 mg/mL stocks) were added after washing the clinical samples (Table S1). The HRP-labelled goat anti-chicken IgG (100 µL/well) was diluted to a concentration of 1:2000 (Table S2).

### 3.5. Cut-Off Values for the NS1-DAS-ELISA

The OD_450nm_ values of 50 clinically negative ChPV samples were determined by the optimal NS1-DAS-ELISA protocol to evaluate the cut-off value of the assay. The mean ($\bar{x}$) OD_450nm_ of the 50 samples was 0.088, the standard deviation (SD) was 0.017, and the critical value was 0.139 according to the formula $\bar{x}$ + 3SD. If the OD_450nm_ of the sample was greater than or equal to 0.139, the sample was considered a ChPV-positive sample. A value less than 0.139 was considered to indicate a negative sample.

### 3.6. Specificity of the NS1-DAS-ELISA

The specificities of the supernatants of LMH cells transfected with the plasmid pBluescript II SK (+)-ChPV (ChPV infectious clone), NDV, MS, FAdV-1, FAdV-4, CIAV, IBV, aMPV, H9N2 subtype AIV, or EDSV and an AGV2-positive sample were tested via the NS1-DAS-ELISA. PBST was used as a negative control. As shown in Table 1, OD_450nm_ values less than 0.139 for ten pathogens (NDV, MS, FAdV-1, FAdV-4, CIAV, IBV, aMPV, H9N2 subtype AIV, EDSV, and AGV2) were considered to indicate a negative sample (Table 1), which suggested that there was no cross-reactivity with NDV, MS, FAdV-1, FAdV-4, CIAV, IBV, aMPV, H9N2 subtype AIV, EDSV, or AGV2 with the NS1-DAS-ELISA method. The NS1-DAS-ELISA detected only the ChPV-positive supernatant (OD_450nm_ = 0.895) (Table 1), indicating that the NS1-DAS-ELISA was highly specific for ChPV detection.

### 3.7. Sensitivity of the NS1-DAS-ELISA

**Figure 2:**
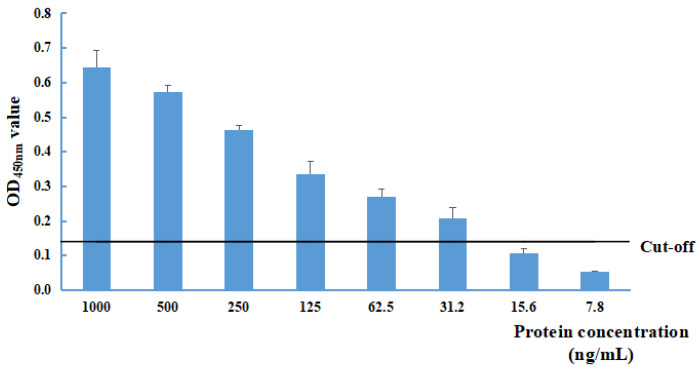
Determination of the limit of detection of the NS1-DAS-ELISA using the recombinant pET32a-NS1 protein antigen. The cut-off value was 0.139.

The sensitivity of the NS1-DAS-ELISA was evaluated with different recombinant pET32a-NS1 protein concentrations (at dilutions of 1000, 500, 250, 125, 62.5, 31.2, 15.6, 7.8, and 0 ng/mL). The limit of detection was 31.2 ng/mL for the recombinant pET32a-NS1 protein (Figure 2).

### 3.8. Repeatability Analysis of the NS1-DAS-ELISA

To evaluate the repeatability of the NS1-DAS-ELISA, the OD_450nm_ was measured for different concentrations of the recombinant pET32a-NS1 protein (at dilutions of 250 ng/mL, 500 ng/mL, 750 ng/mL, and 1000 ng/mL) using the NS1-DAS-ELISA. The CVs of the intra- and interassay data were less than 5%, which indicated that the NS1-DAS-ELISA has good repeatability (Table 4).

### 3.9. Comparison of the NS1-DAS-ELISA and Nested PCR Methods

A total of 192 clinical samples were tested for ChPV by the established NS1-DAS-ELISA and a nested PCR method. A total of 141 samples were ChPV positive according to NS1-DAS-ELISA. Thirty samples were negative and 162 samples were positive according to the nested PCR method (Table 5). The coincidence rate between the NS1-DAS-ELISA and the nested PCR method for ChPV positivity was 87.0% (141/162). The total coincidence rate of the two detection methods was 89.1% (((141 + 30)/192) *%).

## 4. Discussion

Minute virus of mice (MVM), goose parvovirus (GPV), and porcine parvovirus (PPV) belong to the family *Parvoviridae* (www.ictv.global/report/parvoviridae, accessed on 5 December 2023). The NS1 protein is a multifunctional protein in MVM, GPV, and PPV and is associated with the replication of viral DNA, the induction of host cell apoptosis, and the induction of inflammatory reactions [31,32,33,34]. The NS1 protein is a nonstructural protein of ChPV that is highly conserved among ChPV strains [13]. A report by Nuñez et al. [35] showed that ChPV could be isolated through chicken embryo inoculation. We attempted to isolate ChPV in our laboratory according to the method described by Nuñez et al. [35]. In addition, we used different cell lines for ChPV isolation, but all the methods used failed. An appropriate cell line for the isolation of ChPV remains to be identified; therefore, research on the function of the NS1 protein in ChPV is very limited. Although the function of the NS1 protein in ChPV is unclear, the preparation of mAbs against the NS1 protein is a prerequisite for understanding protein function and for detecting ChPV. The mAbs against the NS1 protein of canine parvovirus (CPV) and MVM have been used in ELISA, Western blot, and IFA analyses [36,37]. The mAbs against the NS1 protein of CPV were prepared and used to analyze the distribution of the NS1 protein in cells during the infection phase [36]. A mAb against the NS1 protein was used in IFA and WB analysis by Larsen et al. to determine how MVM localizes to cellular sites of DNA damage [37]. The above data indicate that the development of mAbs against viral proteins is crucial for comprehending viral protein functions and creating specific detection tools for viruses. Two mAbs (1B12 and 2B2) against the NS1 protein of ChPV were successfully produced in this study. The titers of 1B12 and 2B2 were 1.6 × 10^7^ and 4.1 × 10^6^, respectively. The Western blot and IFA analyses indicated that 1B12 and 2B2 could react specifically with the recombinant pET32a-NS1 protein expressed in prokaryotes, as well as the NS1 protein expressed in LMH cells. This study represents a phased achievement, and the prepared mAbs can be used for Western blot and IFA analyses in the future to determine the function of the NS1 protein of ChPV.

Currently, ChPV infection is widespread among commercial chickens, including healthy chickens. ChPV detection has been conducted mainly via nucleic acid testing. However, nucleic acid extraction and PCR-based methods require specialized instruments and strong technical expertise for detection and are not suitable for the rapid analysis of numerous samples on farms. ELISAs are fast and convenient assays that do not involve complex processing of analyzed samples. In addition, ELISAs do not require expensive instruments, are low cost and are easy to perform on farms [38]. In this study, a DAS-ELISA based on a mAb against the ChPV NS1 protein was developed for ChPV detection. The mAb 1B12, which had a high antibody titer, was selected as the capture antibody, and the optimal concentrations of the mAb and polyclonal antibody were determined. The mAb 1B12 only reacted with ChPV according to the results of the NS1-DAS-ELISA, and no cross-reactivity with other pathogens, such as NDV, MS, FAdV-1, FAdV-4, CIAV, IBV, aMPV, H9N2 subtype AIV, EDSV, or AGV2, was observed. These findings indicate that the established NS1-DAS-ELISA has a high specificity for the detection of ChPV. The limit of detection was 31.2 ng/mL for the recombinant pET32a-NS1 protein based on the optimal conditions for the DAS-ELISA. The NS1-DAS-ELISA can be applied to analyze numerous clinical samples and is the first DAS-ELISA for the detection of the ChPV antigen.

A total of 192 samples were analyzed by the NS1-DAS-ELISA and nested PCR method in this study. The results for 21 samples were inconsistent between the two methods; these samples were ChPV-positive according to nested PCR but negative according to the NS1-DAS-ELISA. It is possible that the low concentrations of ChPV in the samples were not detected by the NS1-DAS-ELISA. In addition, there were two limitations associated with the NS1-DAS-ELISA. No ChPV isolates were available for the DAS-ELISA sensitivity test in this study, and the recombinant pET32a-NS1 protein was used as a standard in the sensitivity test, which may lead to bias in the analysis of clinical infection samples by the NS1-DAS-ELISA. Another drawback is that it was unknown whether the NS1-DAS-ELISA could also detect turkey parvovirus (TuPV) because of the high homology of the NS1 protein between TuPV and ChPV.

For the initial establishment of the NS1-DAS-ELISA, chicken polyclonal antibodies and mAbs were used as capture antibodies and detection antibodies, respectively. The OD_450nm_ of the ChPV positive control was less than 0.2, suggesting that the specific binding site between the NS1 protein and the mAb was blocked. This could be due to the preferential binding of polyclonal antibodies to these specific sites, preventing the mAb from binding to the NS1 protein. Therefore, the mAbs and polyclonal antibodies were exchanged for capture antibodies and detection antibodies, respectively, in subsequent experiments, and significant increases in the OD_450nm_ and P/N values of the ChPV-positive control were observed.

In summary, two specific mAbs (1B12 and 2B2) against the NS1 protein of ChPV were screened, and 1B12 was used as the capture antibody for developing an NS1-DAS-ELISA to detect ChPV. This NS1-DAS-ELISA offers a simple and low-cost tool with good specificity and sensitivity for the diagnosis of ChPV infection in chickens.

## Figures and Tables

**Table 1:** Details of pathogens used for testing the specificity of the DAS-ELISA.

Pathogens	Strain Names	Source	Accession No.	Year	Cultures/Tissues	Mean OD_450nm_ Values
NDV	GX6/02	GVRI	-	2002	Chicken embryo allantoic fluid	0.032
NDV	Duck/China/Guangxi19/2011	GVRI	KC920893	2011	Chicken embryo allantoic fluid	0.036
NDV	Chicken/Guangxi/B14/2021	GVRI	-	2021	Chicken embryo allantoic fluid	0.039
MS	PMS156	UC	-	-	MS liquid culture medium	0.067
FAdV-1	GX201802	GVRI	MZ322953	2018	LMH cells cultures	0.06
FadV-1	GX201803	GVRI	MZ322954	2018	LMH cells cultures	0.051
FadV-4	GX2018-07	GVRI	MN577983	2018	LMH cells cultures	0.028
FadV-4	GX2017-06	GVRI	MN577982	2017	LMH cells cultures	0.031
FadV-4	GX2019-09	GVRI	MN577985	2019	LMH cells cultures	0.032
CIAV	GXC060821	GVRI	JX964755	2006	MDCC-MSB1 cell cultures	0.029
IBV	GXIB/02	GVRI	-	2002	Chicken embryo allantoic fluid	0.043
aMPV	MN-10	UC	-	-	Chicken embryo allantoic fluid	0.050
AIV	A/Chicken/Guangxi/LZ066C/2020 (H9N2)	GVRI	-	2020	Chicken embryo allantoic fluid	0.100
AIV	A/chicken/Guangxi/C1228/2015 (H9N2)	GVRI	KX185890	2015	Chicken embryo allantoic fluid	0.067
AIV	A/chicken/Guangxi/C227/2015 (H9N2)	GVRI	KX130848	2015	Chicken embryo allantoic fluid	0.058
EDSV	GEV	GVRI	-	1995	Chicken embryo allantoic fluid	0.045
AGV2	AGV2-GX19010	GVRI	MW404236	2019	Throat and cloaca swab sample	0.017
ChPV infectious clone	GX-CH-PV-21	GVRI	MG602511	2016	LMH cells cultures	0.895

GVRI = Guangxi Veterinary Research Institute, China; UC = University of Connecticut, USA.

**Table 2:** OD_450nm_ values of the isotyping assay for mAbs (1B12 and 2B2).

mAbs	IgG1	IgG2a	IgG2b	IgG2c	IgG3	IgM	Kappa	Lambda
1B12	1.023	0.112	0.088	0.089	0.093	0.130	0.521	0.076
2B2	0.744	0.098	0.201	0.176	0.101	0.128	0.634	0.099

**Table 3:** The OD_450nm_ values of 1B12 and 2B2 used as the capture antibodies in the DAS-ELISA.

mAbs	Mean OD_450nm_ Values			
	Recombinant pET32a-NS1 Protein	PBS Control	ChPV Infectious Clone	Negative LMH Cells
1B12	1.025	0.056	0.806	0.069
2B2	0.648	0.073	0.450	0.102

**Table 4:** Intra- and interassay repeatability of the NS1-DAS-ELISA.

Concentrations of Recombinant pET32a-NS1 Protein	Intra-Assay			Inter-Assay		
	Mean OD_450nm_	SD	CV	Mean OD_450nm_	SD	CV
250 ng/mL	0.50	0.018	3.5%	0.511	0.017	3.3%
500 ng/mL	0.51	0.023	4.6%	0.492	0.012	2.4%
750 ng/mL	0.70	0.027	3.8%	0.643	0.032	4.9%
1000 ng/mL	0.77	0.018	2.3%	0.671	0.032	4.7%
0 ng/mL	0.06	0.002	3.4%	0.069	0.003	4.9%

**Table 5:** Comparison of the NS1-DAS-ELISA and nested PCR for the detection of ChPV in clinical samples.

NS-DAS-ELISA		**Nested PCR**		
		**Positive**	**Negative**	**Total**
	Positive	141	0	141
	Negative	21	30	51
	Total	162	30	192

## Data Availability

All the data are contained in the manuscript, figures, and Supplementary Tables.

## Supplementary Materials

The following supporting information can be downloaded at: https://www.mdpi.com/article/10.3390/pathogens13030221/s1. Table S1: The OD_450nm_ values of the different concentrations of the capture antibody and detection antibody. Table S2: The OD_450nm_ values of the different concentrations of HRP-labelled goat anti-chicken IgG. The results show the mean OD_450 nm_ values ± SDs.
