# Supplementary material for: Development of a Double-Antibody Sandwich ELISA Based on a Monoclonal Antibody against the Viral NS1 Protein for the Detection of Chicken Parvovirus
Source: Pathogens. 2024 Mar 1;13(3):221. doi: 10.3390/pathogens13030221 (PMC10976255; doi:10.3390/pathogens13030221)
Supplement: Supplementary file 1 [file pathogens-13-00221-s001.zip › pathogens-2867553-supplementary.pdf]

Supplementary Material

Table S1. $OD_{450\ nm}$ values of the different concentrations of the capture antibody and detection antibody.

Concentration of capture antibodies		Concentration of detection antibodies				
		1:1000	1:2000	**1:4000**	1:8000	1:16000
1:1000	Positive samples (OD_{450nm})	0.953±0.03	0.846±0.03	0.843±0.04	0.755±0.08	0.747±0.08
	Negative samples (OD_{450nm})	0.106±0.03	0.084±0.01	0.074±0.01	0.072±0.01	0.066±0.01
	P/N	9.0	10.1	11.4	10.5	11.3
**1:2000**	Positive samples (OD_{450nm})	0.806±0.01	0.779±0.01	**0.752±0.01**	0.641±0.01	0.608±0.01
	Negative samples (OD_{450nm})	0.081±0.01	0.075±0.01	**0.060±0.01**	0.057±0.01	0.052±0.01
	P/N	9.9	10.4	**12.5**	11.2	11.7
1:4000	Positive samples (OD_{450nm})	0.649±0.02	0.636±0.07	0.606±0.04	0.524±0.04	0.507±0.07
	Negative samples (OD_{450nm})	0.076±0.01	0.064±0.01	0.063±0.01	0.053±0.01	0.051±0.01
	P/N	8.5	9.9	9.6	9.9	9.9
1:8000	Positive samples (OD_{450nm})	0.560±0.08	0.484±0.07	0.454±0.05	0.416±0.03	0.386±0.01
	Negative samples (OD_{450nm})	0.069±0.01	0.066±0.01	0.056±0.01	0.045±0.00	0.043±0.00
	P/N	8.1	7.3	8.1	9.2	9.0

Bold indicates the optimal concentrations of the capture antibody and detection antibody for the NS1-DAS-ELISA method. The P/N value represents the OD_{450nm} value of the samples (P value)/$OD_{450\ nm}$ value of the negative control (N value), and a P/N value greater than 2.1 indicates a positive result.

Table S2. OD_{450nm} values of the different concentrations of HRP-labelled goat anti-chicken IgG.

Concentrations of HRP-labelled goat anti-chicken IgG	Positive samples (OD_{450nm})	Negative samples (OD_{450nm})	P/N
1:500	1.396±0.11	0.156±0.01	8.946
1:1000	1.020±0.06	0.111±0.02	9.189
**1:2000**	**0.770**±0.04	**0.065**±0.00	**11.938**
1:4000	0.432±0.02	0.046±0.01	9.380
1:8000	0.200±0.00	0.047±0.01	4.290

Note: Bold indicates the optimal concentrations of HRP-labelled goat anti-chicken IgG for the NS1-DAS-ELISA; the P/N value represents the OD_{450nm} value of the samples (P value)/the OD_{450nm} value of the negative control (N value); a P/N value greater than 2.1 indicates a positive result.
